# Arrayed electro-optic modulators for novel WDM multiplexing

**DOI:** 10.1038/s41598-024-62755-z

**Published:** 2024-05-24

**Authors:** Behrang Hadian Siahkal‑Mahalle, Kambiz Abedi

**Affiliations:** Faculty of Electrical Engineering, Shahid Beheshti University, Tehran, Iran

**Keywords:** Wavelength-division multiplexing (WDM), Arrayed electro-optical modulators, Indium tin oxide (ITO), Optics and photonics, Integrated optics, Optoelectronic devices and components

## Abstract

In this paper, a novel silicon-on-chip integrated 4 × 1 wavelength division multiplexing (WDM) multiplexer has been developed. This is the first time that the multiplexer design incorporates arrayed electro-optical modulators with crosstalk cancellation. The design utilizes two types of electro-optic modulators in each channel. The first modulator, based on 1D-PhCNBC, extracts the desired wavelengths from the WDM spectrum. The second modulator, based on coupled hybrid plasmonics, acts as a switch to eliminate crosstalk of the desired optic wavelength signal at the multiplexer output. By combining the advantages of electro-optical modulators and crosstalk cancellation techniques, we anticipate that our proposed design contributes to the advancement of WDM multiplexing technology and facilitates the implementation of efficient and compact optical communication systems. Additionally, this synergy enables enhanced performance, reduced signal interference, and improved signal quality, leading to more reliable and high-speed data transmission in optical networks. The functionality of the device is theoretically simulated using 3D-FDTD (Finite-Difference Time-Domain) method.

## Introduction

The rapid growth of multimedia communications has resulted in a significant increase in telecommunication network traffic, highlighting the urgent need for new high-capacity networks. Therefore, one of the primary concerns in communication networks is to achieve high bandwidth and optimize its utilization. Optical systems have emerged as the preferred choice for telecommunication networks due to their advantages over electrical systems. However, certain devices such as switches still rely on relatively slow electrical technology, despite the transition to optical transmission lines.

In optical telecommunication systems, the widely used time-division multiplexing (TDM) method heavily relies on electrical circuits for signal multiplexing (MUX) and demultiplexing (DEMUX). This necessitates the use of optical-electrical converters, MUXs, DEMUXs, and electrical-optical converters within TDM systems. The limitations of electrical circuits result in a drop in network efficiency, leading to fundamental problems. As a result, the design of devices used in optical technologies and the need for optical MUXs have become crucial for improving system efficiency.

Optical MUXs, combined with wavelength-division multiplexing (WDM) technology, enable telecommunication systems to combine hundreds or even thousands of different optical wavelengths in the transmitter and transmit them through an optical fiber, where they are separated at the receiver. This allows for the optimal utilization of all optical bandwidths^1,2^. Various designs for optical MUXs in silicon (Si) photonics have been proposed, including Si micro-ring MUXs^3^, Si-array waveguide grating (AWG) MUXs^4,5^, multimode interference (MMI) MUXs^6^, and Si Mach–Zehnder switches MUXs^7^. However, integrating these MUXs with other nanoscale optical devices presents significant challenges mainly due to their large footprints.

To address these challenges, photonic crystals (PCs), which are alternate structures made of dielectric materials, have been utilized. PCs control light propagation in specific directions and wavelengths while preventing propagation in other directions and wavelengths. This feature allows for effective control of light propagation in short-distance optical devices, making it possible to create ultra-compact devices^8–11^. Consequently, the development of ultra-small MUXs based on PC structures has gained attention. One-dimensional photonic crystal structures, in particular, offer attractive features such as an ultra-compact footprint, high-quality (Q) factor, and seamless integration with optical waveguides, surpassing their 2D and 3D counterparts^12–17^.

This paper presents a 4 × 1 hybrid electro-optical MUX that utilizes two hybrid modulators in each channel to cover dense wavelength-division multiplexing (DWDM) wavelengths. The first modulator in each channel is based on a 1-dimensional photonic crystal nanobeam cavity (1D-PhCNB) that incorporates indium tin oxide (ITO). The second electro-optic modulators in each channel also utilize ITO and are based on surface plasmon polariton (SPP)^18^. The proposed MUX design offers a highly compact footprint and low energy consumption, thanks to the well-designed modulators^19,20^.

Furthermore, the electrical tunability of ITO enables multiplexing on all wavelengths of the DWDM. To address one of the most significant challenges in MUXs, namely crosstalk (the overlap of adjacent spectra), the structured design incorporates a second modulator in each channel. This modulator acts as a shield, preventing the output spectra of adjacent channels from interfering with each other until the full transfer of the output spectrum of a channel is achieved. The second modulator achieves this by switching between on and off modes, utilizing the SPP feature within the epsilon-near-zero region for efficient multiplexing.

This article presents the general structure of the proposed MUX and describes its functionality. The third section discusses simulations and discussions related to the operation and cancellation of crosstalk. Finally, the conclusion summarizes the findings of the study.

## Designing the device

**Figure 1:**
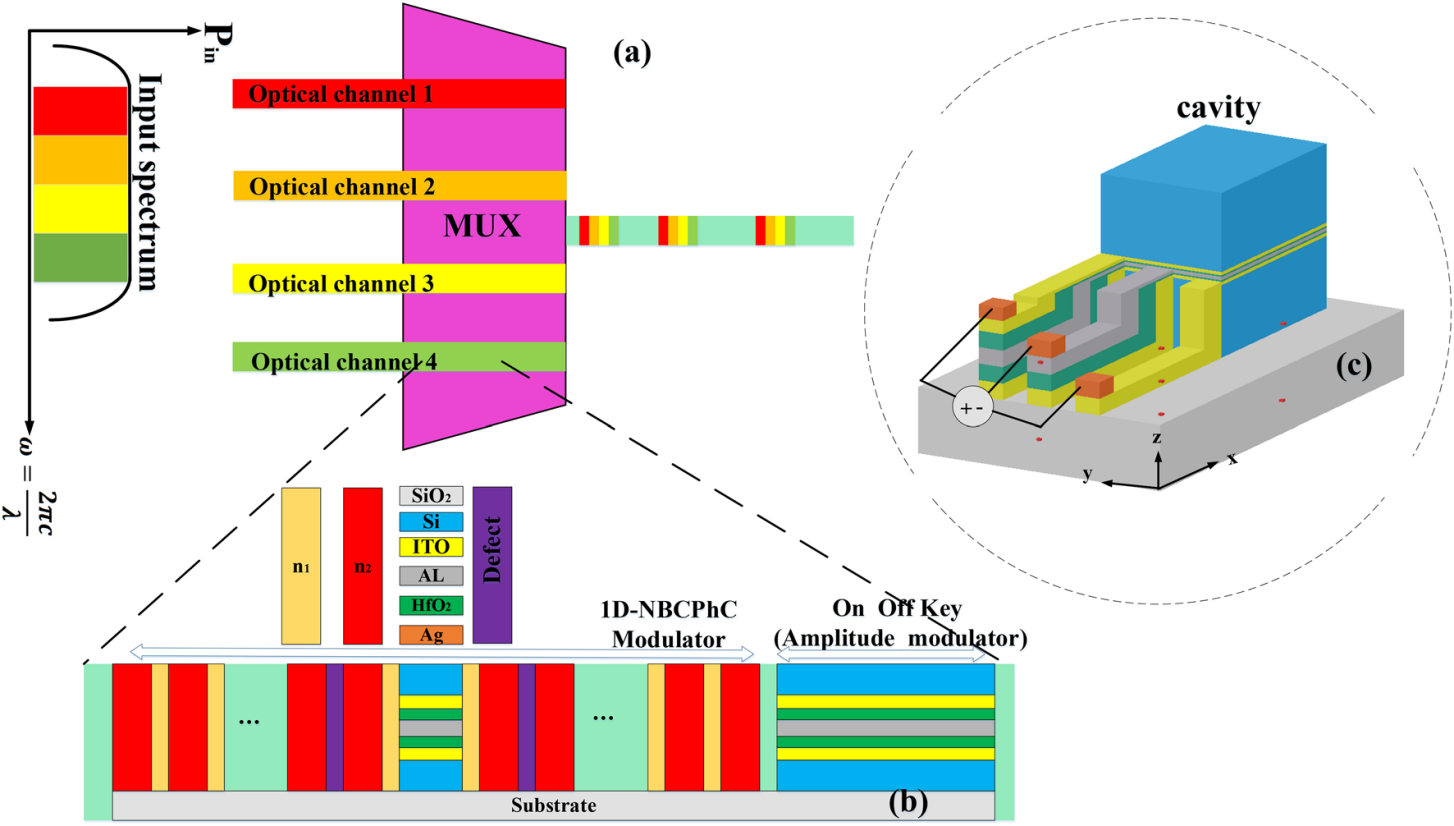
(**a**) The main idea used in designing MUX. (**b**) The general structure of the modulators used in each channel. (**c**) The biased method of both modulators of each of the 4 channels.

The objective of this paper is to design and simulate a 4 × 1 MUX (multiplexer) that minimizes crosstalk and optimizes bandwidth utilization in WDM (Wavelength Division Multiplexing) technology. The optical system depicted in Fig. 1 forms the basis of the design and relies on electro-optic modulators. The biasing method for the modulators in each of the 4 channels is illustrated in Fig. 1C^21^.

**Figure 2:**
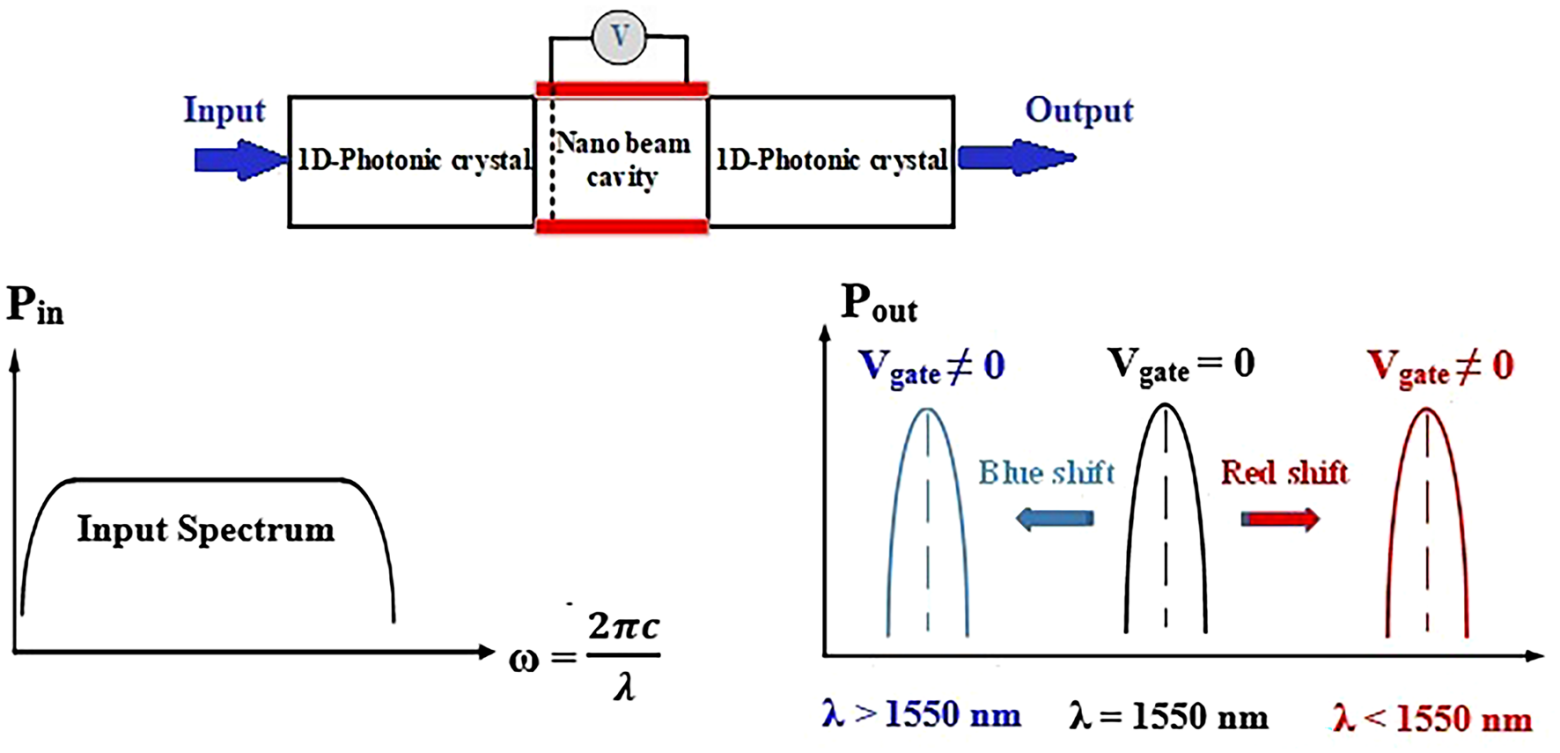
The main idea of the use of the 1D-NBCPhC electro-optical modulator in the design of the MUX.

In each channel's design, two types of modulators are utilized. The first type, referred to as Modulator 1 (Mod.1), acts as a switch and is a 1-Dimensional Nanobeam Cavity Photonic Crystal (ID-NBCPhC) electro-optical modulator. It is positioned at the input port of each MUX channel and is responsible for extracting standard WDM telecommunication channels from the input optical spectrum. Mod.1 functions as a low-pass filter, with its central frequency aligned with the frequency of the PhCNB. After entering the first type modulator, the spectrum oscillates between two parts of a one-dimensional photonic crystal located on either side of the cavity, which act as mirrors. This action results in a narrow spectrum being filtered out from the input spectrum or from the same cavity (Fig. 2).

The cavity's main structure is composed of indium tin oxide (ITO), which, similar to polymers^22^, exhibits a change in refractive index when a voltage is applied. Consequently, the output wavelengths differ between biased and non-biased modes due to the refractive index change. Applying bias to most polymers increases the refractive index and causes a redshift in the selected wavelength. However, in this design, polymers are not used due to their destructive thermal effects on modulator performance, as well as integration challenges with other optical devices^23^. By reducing the effective refractive index of the cavity in Mod.1 through the voltage-induced decrease in ITO refractive index, a blueshift is observed. Since the objective of this design is to cover all the spectra used in DWDM (Dense Wavelength Division Multiplexing), this is accomplished by introducing defects in the photonic crystal sections of the device channels.

**Figure 3:**
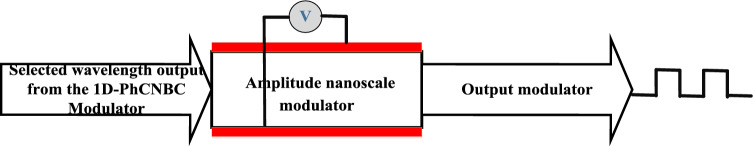
The main idea used in the design of the second type modulator of each (de)multiplexer channels.

After emitting light with the desired wavelength in the waveguide before exiting each channel and reaching the MUX output, another electro-optical modulator, based on coupled hybrid plasmonics, is employed. This second modulator in each channel ensures that the selected wavelength is properly placed among the input sources at the output port of the MUX, without any crosstalk. Consequently, in the design of this modulator, known as Modulator 2 (Mod.2), minimal losses are required in the ON mode (light transmission), while maximum loss is needed in the OFF mode (absorbing input light). A trade-off must be made between insertion loss and extinction ratio in the modulator to achieve the best possible performance. ITO is also the primary material used in Mod.2. Unlike Modulator 1, ITO in Mod.2 enters the ENZ (epsilon-near-zero) region through biasing, leading to an increase in the imaginary part of the refractive index, resulting in increased loss and wavelength absorption without propagation. The performance of Mod.2 is illustrated in Fig. 3.

The most significant design parameters in both types of modulators are similar, with the main difference lying in the photonic crystal component, which is not utilized in Mod.2. The cavity of Mod.1 and the structure of Mod.2 both consist of two metal–oxide–semiconductor (MOS) stacks, with ITO located in the central part of each stack. The utilization of these two ITO-included stacks connected by an AL (Aluminum) layer in both modulators confines the light to a small volume and enhances the interaction between light and material. In Mod.1, the cavity length is 200 nm, with a = 158.114 nm, 10 dielectric pairs of n1, and 9 dielectric pairs of n2 on each side of the cavity. The structure's height is h = 500 nm, and its width is w = 300 nm. Within the cavity, the HfO_2_, ITO, and AL layers in each stack have thicknesses of 5 nm, 10 nm, and 10 nm, respectively. A TM-like fundamental 1st order mode stimulates the 1D-PC along the Z axis, while the device length range falls along the X axis. In Mod.2, the layer layout and dimensions are similar to Mod.1, with only the length increasing to 888.57 nm. The total structural length of Mod.1 is 6308 nm, and both types of modulators have been integrated on a silicon-on-insulator (SOI) background with a buried oxide layer of 1 µm.

### Using ITO as the main component of the design

In recent years, extensive research has focused on developing new material platforms for fabricating optical plasmonic devices^24–31^. Indium Tin Oxide (ITO) is one such material, functioning as a transparent conductive oxide with a controllable optical response through the application of bias. By adjusting the concentration of free carriers within the range of 10^19^ cm^−3^–10^21^ cm^−3^, ITO exhibits quasi-metal behavior in the near-infrared wavelength range, enabling the design of subwavelength light^30^. Unlike common metals, which possess fixed optical characteristics, ITO's permittivity can be modified through doping and fabrication processes, making it advantageous for designing plasmonic and nanophotonic devices^24,30,32^. Additionally, ITO is compatible with CMOS technology and exhibits high resistance to thermal changes.

When ITO is subjected to external bias, it leads to changes in the carrier concentration (n_acc_), subsequently affecting its plasma frequency (w_p1_) and resulting in observed changes in permittivity. These changes can be described using the Drude model equation:

1 $$n_{acc} = n_{0} + \frac{{\varepsilon_{0} \cdot \kappa_{die} \cdot V_{g} }}{{e \cdot t_{die} \cdot t_{acc} }}$$

2 $$\varepsilon_{acum} (\omega ) = \varepsilon_{1} + j\varepsilon_{2} = \varepsilon_{\infty } \omega_{pl}^{2} /\omega (\omega + i\gamma )$$

Here, n_0_ represents the initial carrier concentration, $\varepsilon_{0}$ denotes vacuum permittivity, *k*_*die*_ is the DC permittivity of the insulator, *V*_*g*_ represents the external bias, *e* is the fundamental charge, t_die_ is the dielectric thickness, t_acc_ is the thickness of the accumulation layer, $\omega_{pl}^{2} = n_{c.c} e^{2} /\varepsilon_{0} m_{eff}$ represents the plasma frequency, $\varepsilon_{\infty }$ is the background permittivity, $\gamma$ is the collision frequency, and $m_{eff}$ is the effective mass of an electron. The thickness of the accumulation layer (active layer), typically ranging from 1 to 3 nm, can be explained by Fermi screening theory^30–32^.

In the case of Modulator 1 (Mod.1), since the shift in the peak wavelength of input light occurs before reaching the ENZ (Epsilon-Near-Zero) region, there is no need to apply high voltages to reach this region. Mod.1 is primarily used for selecting specific wavelengths from the input spectrum, resulting in minimal changes to the imaginary part of the refractive index at low applied voltages, allowing the losses to be neglected.

In Modulator 2 (Mod.2), the zero-bias mode allows the input light spectrum to pass through with minimal loss. However, by applying bias, ITO enters the ENZ region, leading to an increase in the absorption coefficient and consequently causing maximum losses in the input spectrum. By designing the second type of modulator, employing the interaction of the ENZ effect and field engineering, we can achieve the highest level of material-light interaction while simultaneously maximizing the off-mode loss (bias applied). This design results in modulator integration with improved extinction ratio (ER) and insertion loss (IL), nanoscale footprint, and power consumption at the femtojoule (fJ) level. It should be noted that the voltage applied to the second type of modulator is sufficient for entering the ENZ region, causing significant changes in the real and imaginary parts of the refractive index compared to Mod.1.

**Figure 4:**
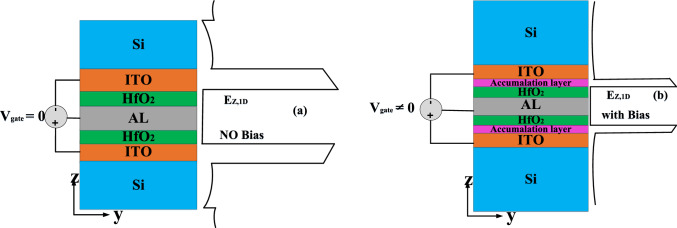
Field intensity profiles within the cavity for (**a**) no bias mode, (**b**) in the mode of applying bias and forming an accumulation layer.

Applying bias to ITO in each modulator results in the formation of an accumulation layer adjacent to the dielectric layer, where the concentration of free carriers (electrons) is significantly higher compared to other parts of ITO (Fig. 4). This leads to a decrease and increase in the real and imaginary parts of the refractive index, respectively.

The fabrication of our multilayer structure requires electron-beam-lithography patterning in combination with the lift-off process. High-resistivity hafnium dioxide (HfO_2_) and indium tin oxide (ITO) layers are deposited through RF magnetron sputtering. The carrier concentration of the as-deposited ITO can be controlled by tuning the oxygen concentration during deposition. Moreover, hydrogenated amorphous polysilicon is deposited via low temperature-PECVD and serves as the top high-index dielectric layer. To minimize the disturbance to the optical mode, electrical contacts will be made through Au pads evaporated onto narrow strips of pre-deposited Al and ITO films. These strips have been strategically extended several hundred nanometers away from the waveguide. For the one-dimensional crystal photo grid, the usual manufacturing methods can also be used.

## Simulation results of the proposed design

To calculate the output wavelength of each channel in the MUX, Maxwell’s equations related to the structure need to be written in order to determine the field values through numerical methods. In this study, the Finite-Difference Time-Domain (FDTD) method has been used to improve computation accuracy and optimize time efficiency. To achieve the cavity mode, an electrical dipole source in the z-direction is placed inside the cavity of Mod.1, which creates a TM-like fundamental 1st order mode within the cavity. The electrical field of the TM mode along the X-axis in the propagation waveguide and the active region is confined by a lower refractive index in the cavity. The feedback from the mirror parts allows for the confinement of light in the center of the cavity along the propagation direction. It is worth noting that the cavity serves as a place to store energy and observe field profiles, and the field intensity is highest near the center of the cavity.

**Figure 5:**
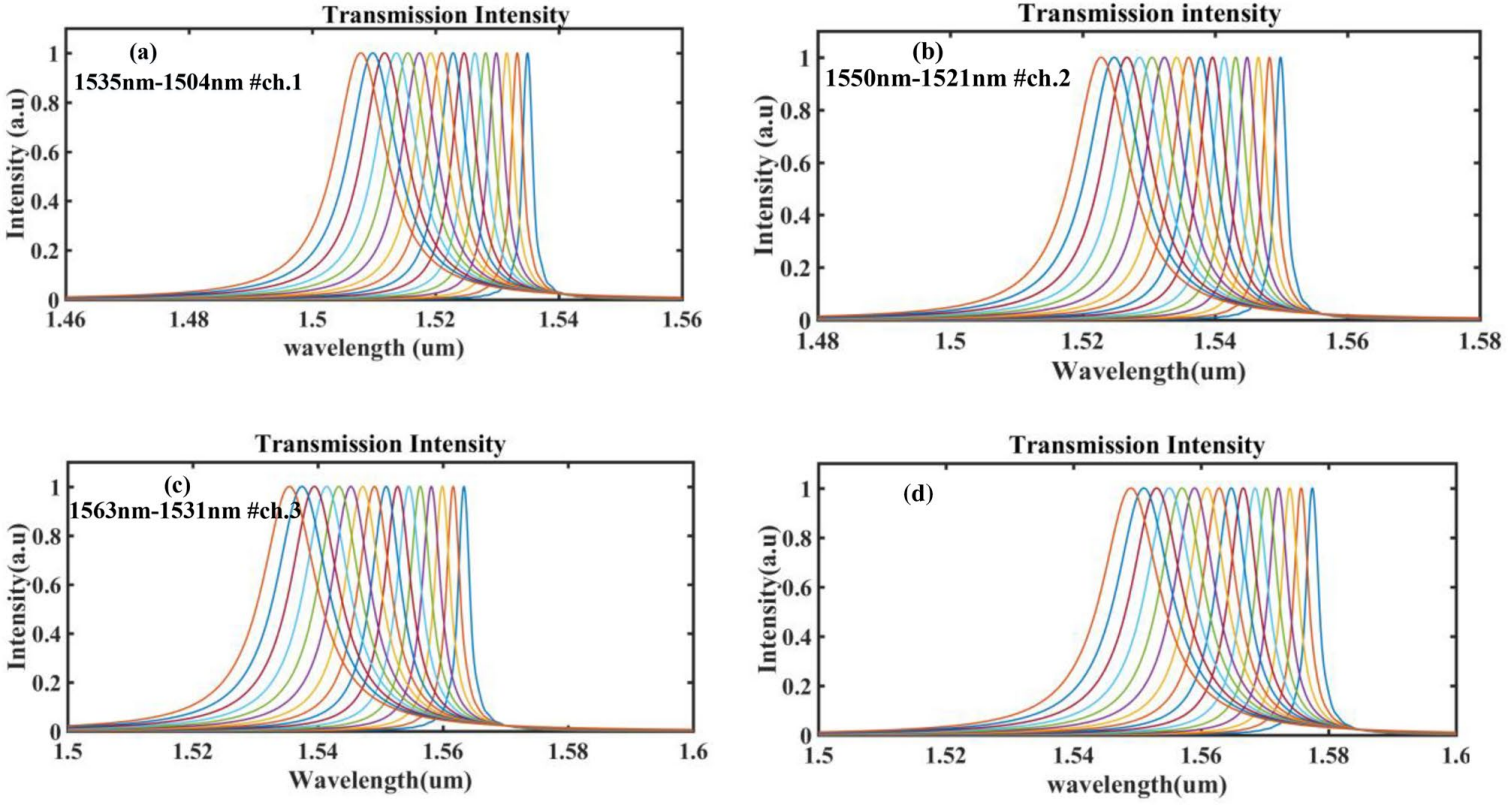
(**a**–**d**) The results of changes in carrier concentration on wavelength adjustment, Δλ, due to the application of voltages from zero to 0–0.3 V with variation of 0.02 V for each of the four channels cavity.

The resonant shift (Δλ adjustment) occurs through changes in the carrier concentration, which in turn results in a change in the real part of the effective refractive index (∆*n*_*eff*_) in Mod.1. The changes in carrier concentration for wavelength adjustment (∆λ) by applying voltages ranging from 0 to 0.3 V with a step of 0.02 V for each of the four channels' cavities are presented separately in Fig. 5.

The designed structure can cover all standard Dense Wavelength Division Multiplexing (DWDM) channels. In the OFF state (bias applied) of Mod.1, a blue shift in the cavity resonances occurs due to the reduction of the effective refractive index. Although there is an increase in the imaginary part of the refractive index, resulting in minimal losses, these losses can be computed using Kramers–Kronig relations. The concentration level of the carrier, adjusted through voltage application, is the adjustable parameter in this structure.

**Figure 6:**
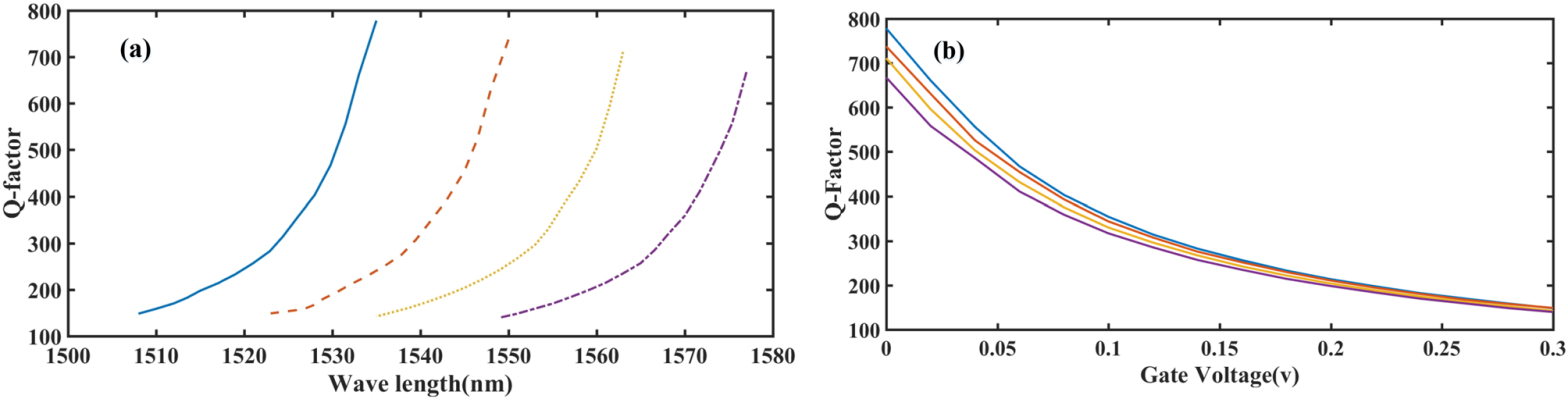
(**a**) Changes Q factor for 4 channel. (**a**) In terms of variations in the wavelength range of each channel and (**b**) In terms of bias changes.

The quality factor (Q factor) can be computed using the low-Q cavity method, where the Q value is measured through the field Fourier transform by finding resonant wavelengths (λ_r_) from signals and determining the full width at half maximum (FWHM, Δλ) from the resonant peaks (Q = λ_r_/Δλ). The Q values for the PCNB cavities in biased and unbiased modes, based on DWDM wavelengths, are shown in Fig. 6 for all four channels. It should be noted that a high Q factor is obtained when the optical mode resonance in the cavity perfectly matches the optical mode in the photonic mirror. The Q factor value with no bias applied is higher than the mode with bias, mainly due to the increase in optical FWHM.

**Figure 7:**
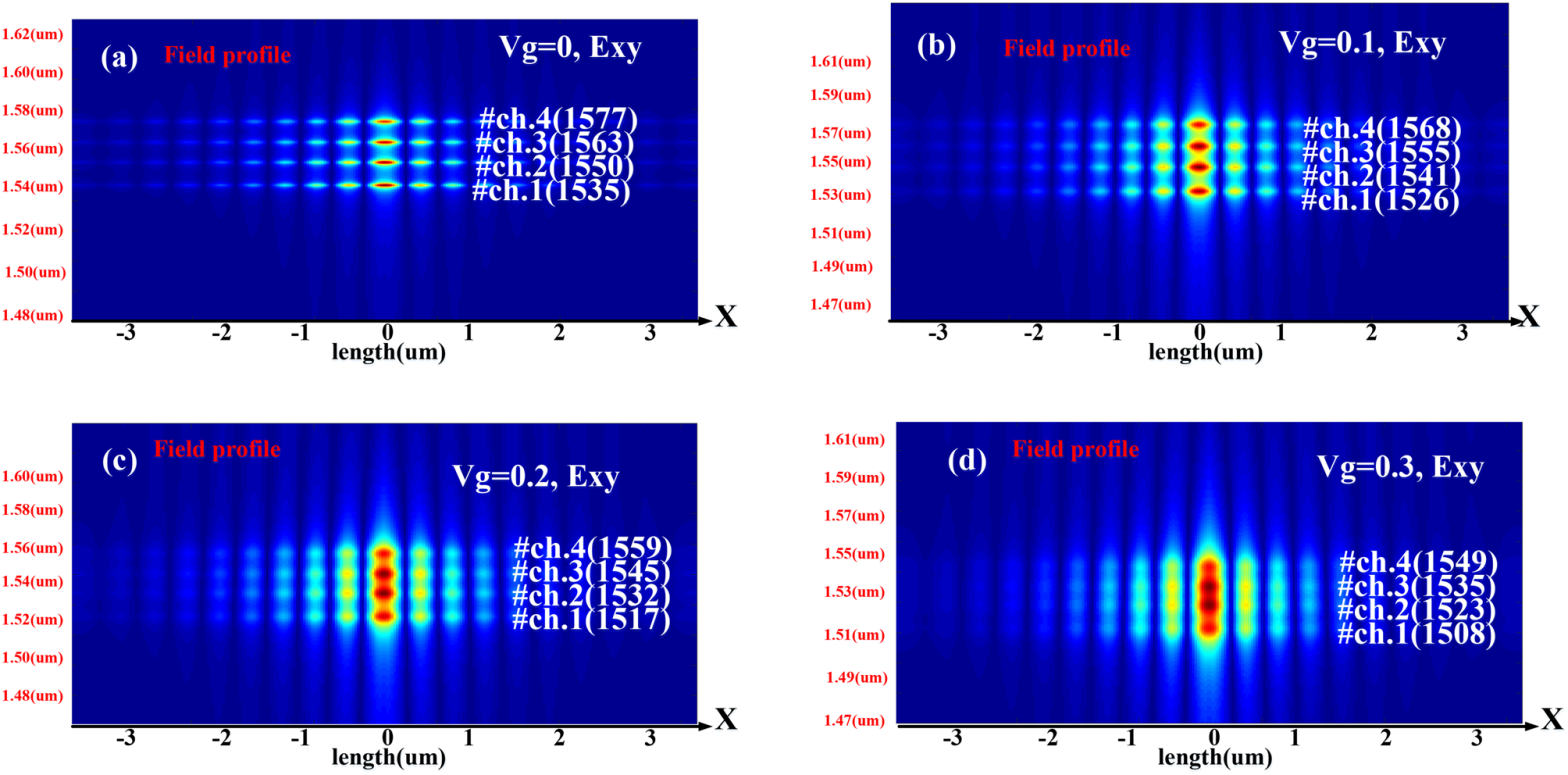
(**a**–**d**) Crosstalk between 4 channels of the device for different voltages.

In the proposed structure, crosstalk occurs between adjacent channels and, in more severe cases, even non-adjacent channels when voltages greater than 0.3 V are applied, especially without using Mod.2. This destructive phenomenon dramatically reduces the efficiency of the structure (Fig. 7), and crosstalk is the amount of energy leakage from one channel into its adjacent or non-adjacent channels. In addition, by applying voltages higher than 0.3 V, the loss amount increases in structure due to the increase in imaginary part of the refractive index and thus the increase in absorption.

**Figure 8:**
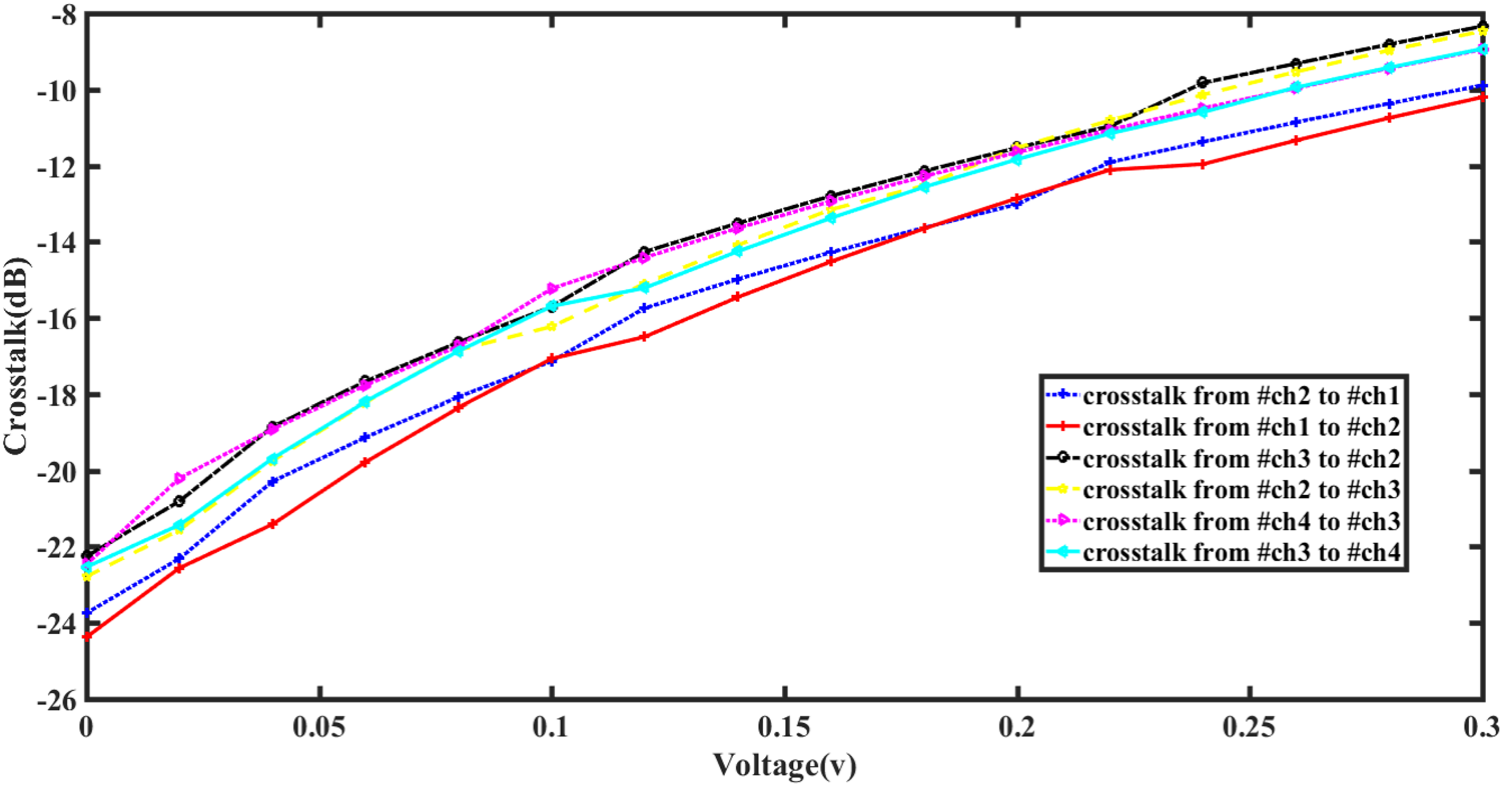
The crosstalk between adjacent channels in terms of the voltage applied to the Mod.1.

To compute the amount of crosstalk between adjacent channels, a transmission diagram is used, and the distance between the continuation of the first channel and the peak of the adjacent channel is determined as the crosstalk amount. The minimum amount of crosstalk in the proposed design, without bias and without inserting Mod.2 at the end of the MUX channel, is equal to − 24.3770 dB from channel 2 to channel 1. The crosstalk between adjacent channels based on the voltage applied to Mod.1 is presented in Fig. 8.

The crosstalk amount between adjacent channels up to 0.3 V applied is at an acceptable level for telecommunication systems. However, applying voltages higher than 0.3 V increases the loss amount in the structure due to the increase in the imaginary part of the refractive index and, consequently, the increase in absorption. The application of the voltage up to 0/3 V, on the other hand, is due to the fact that by applying the voltages with the steps of 0.02 V to the Mod.1, all of the DWDM channels were covered, which this spectral coating can be seen in Fig. 6a.

**Figure 9:**
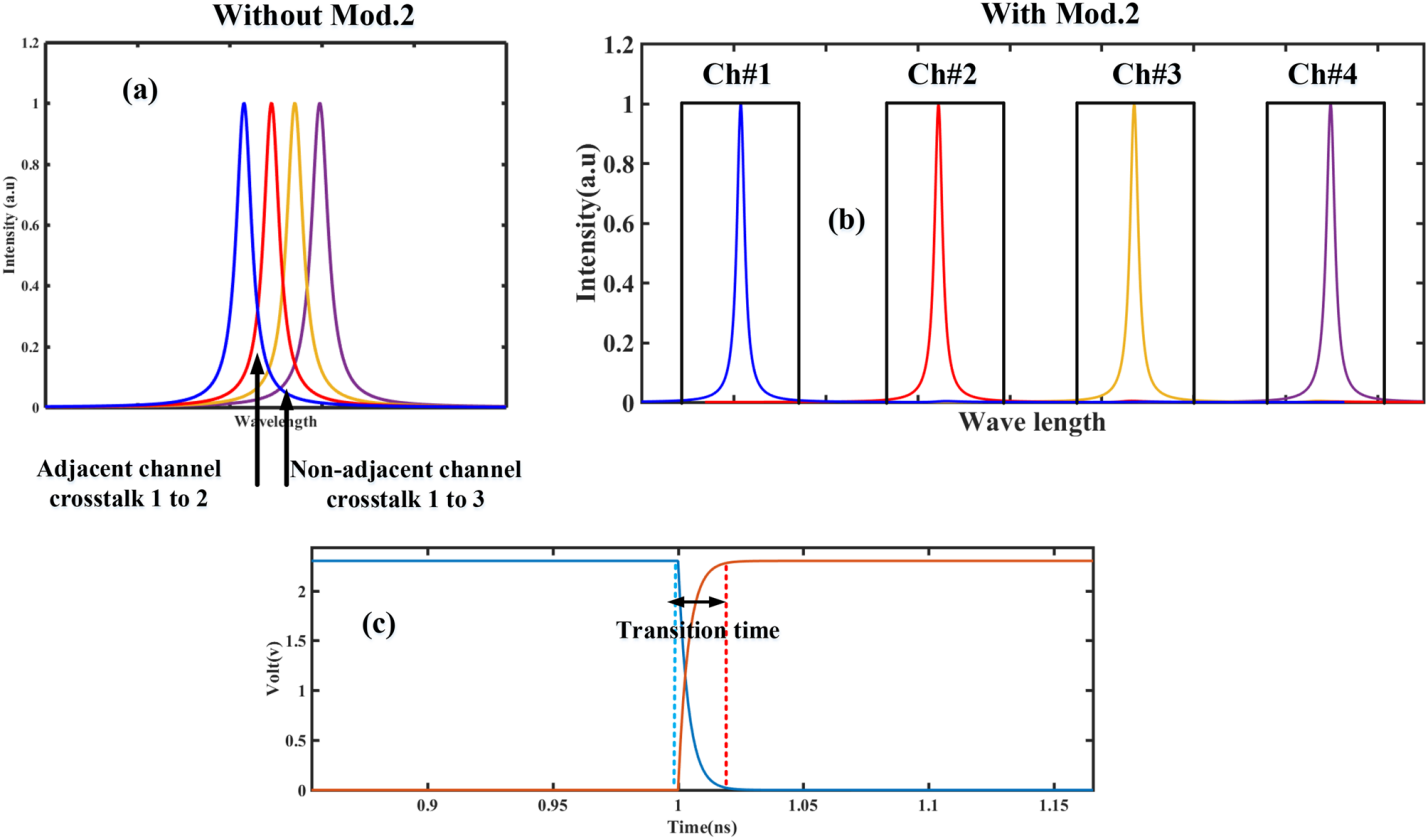
(**a**) crosstalk between adjacent and non-adjacent channels in the case where no Mods.2 are used. (**b**) Removing the crosstalk when using Mods.2. (**c**) Non-ideal performance of the Mods.2 and transition time in switching off and on states.

To eliminate crosstalk in the proposed MUX, Mod.2 is responsible for this task. Mod.2 acts as a switch, turning on and off to prevent interference between adjacent channels. Figure 9a shows the overlap between the outputs of four adjacent channels for an applied voltage of 0.3 V, which represents the worst-case scenario. If the signal of the first channel is placed on its output while the Mod.2 of this channel is switched on and the Mods.2 of the other three channels are turned off (preventing signal propagation), the signal of the first channel is transmitted to the MUX output without overlapping with the outputs of the other three channels (Fig. 9b).

**Figure 10:**
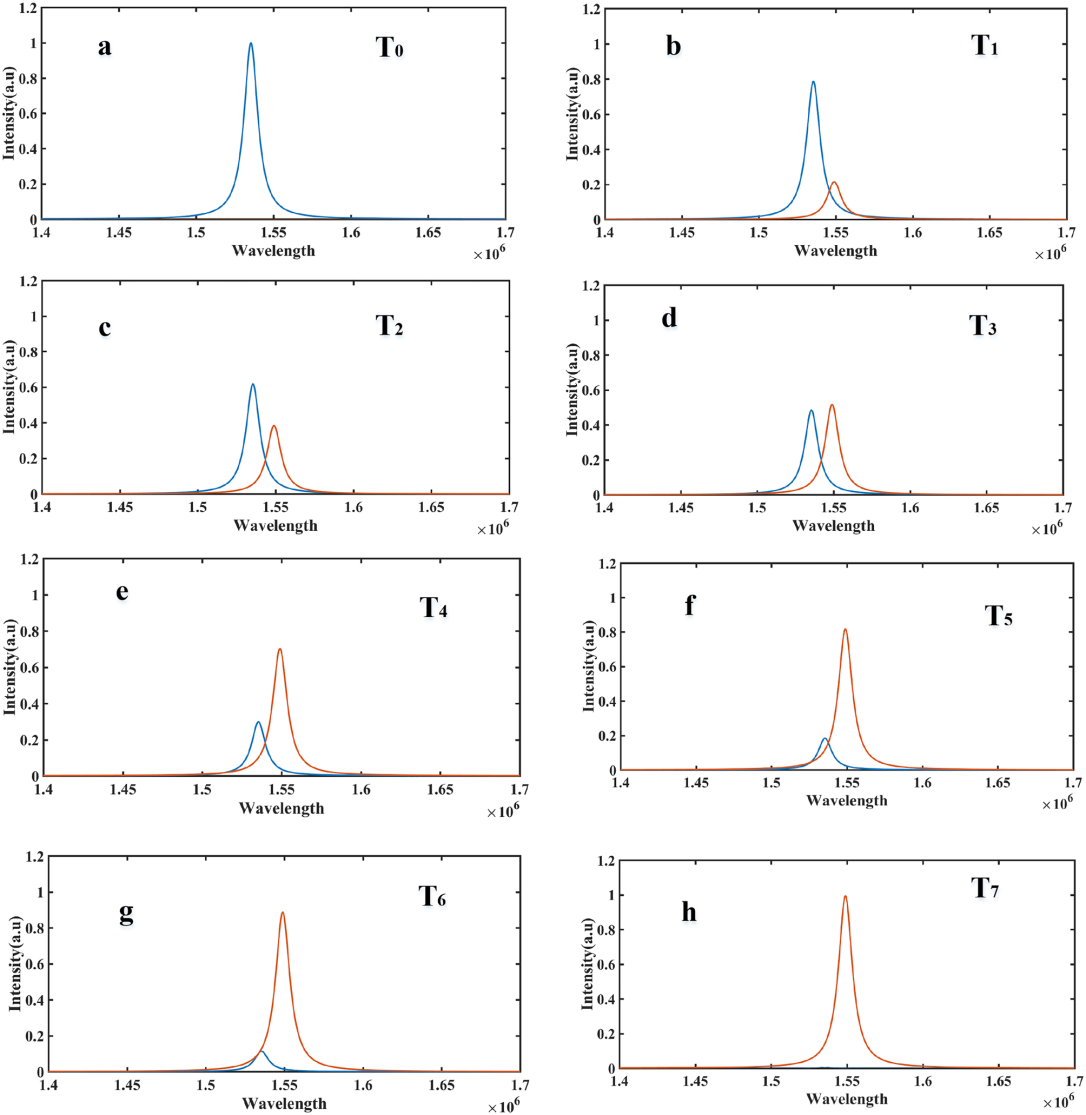
(**a**–**h**) How to create and remove a crosstalk at transition time in two adjacent channels 3 and 4.

The RC time constant of the MOS junction is an important factor to consider as it affects the ideal function of second type modulators. This is illustrated by the dashed line in Fig. 9c^21^. Instead of instantaneous ON and OFF time switching of Mods.2 in each channel, there are delays represented by τ = RC (transition occurs during this time). The output follows the relation $y(t) = y(0) + (y(\infty ) - y(0))e^{{ - \frac{t}{\tau }}}$. The capacitance (C) of the MOS junction can be computed as $C \approx C_{ins} = \frac{{\varepsilon_{ins} }}{{t_{ins} }}A$. Where,$A = H \times L$ represents the cross-section, $\varepsilon_{ins}$ is the DC permittivity, and $t_{ins}$ is the thickness of the insulation layer. On the other hand, the resistance (R) can be obtained as the combination of ITO and AL resistors in series. In the papers studied, it has been approximately 500 Ω, but it could be improved to 200 Ω^21,26^. Due to this limitation, the overlap of channel signals occurs for a short time and fades out after the switching time. Figure 10 shows the result of the overlap and its removal by Mods.2 when applying a voltage of 0.3 V to the Mods.1 of channels 3 and 4, which have the worst crosstalk. Based on these discussions, it can be concluded that the proposed scheme completely eliminates crosstalk in nonadjacent channels. In the case of adjacent channels, there is only a short time interval equal to the switching time between the ON and OFF states of Mods.2 in adjacent channels (Fig. 10).

**Figure 11:**
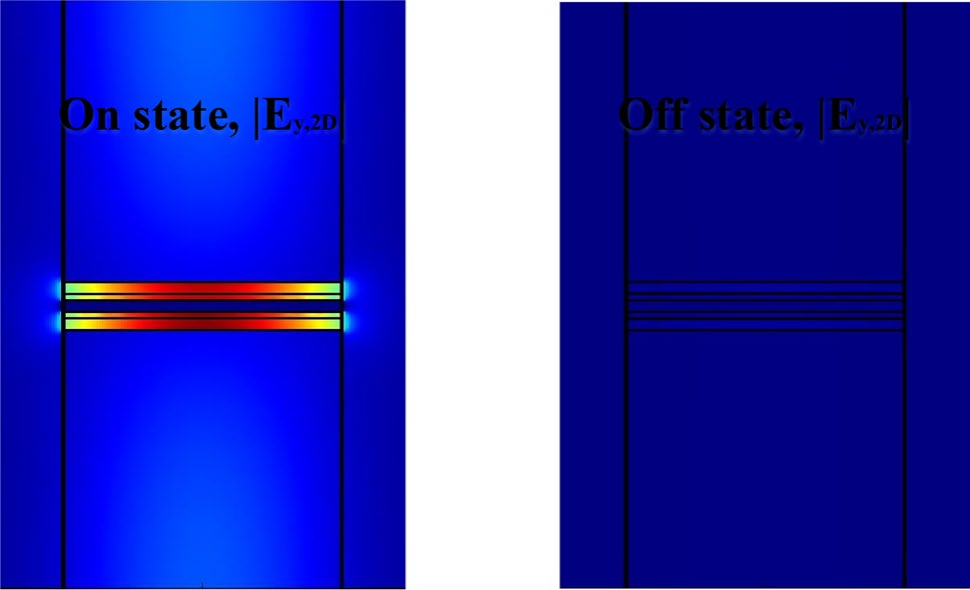
Normalized mode profile of the cross section in the Mod.2 at ON-state and OFF-state.

From a physical perspective, Mod.2, working in the epsilon-near-zero (ENZ) region and providing a plasmonic state during bias application, increases the absorption intensity, resulting in almost no light being transmitted to the output. However, this non-transference does not happen instantaneously but instead has a time constant. In the proposed structure, the highest absorption occurs in the OFF state at 2.3 V. By considering this voltage as logic 1 and the non-bias state where the modulator emits the input light as logic 0, the desired wavelength can be obtained at the MUX output for each instant with the composition of the following combinations: (0,1,1,1), (1,0,1,1), (1,1,0,1), and (1,1,1,0) (Fig. 11).

Considering that the proposed MUX is based on two types of modulators, the performance of the device is affected by parameters such as insertion loss (IL), extinction ratio (ER), bandwidth, power consumption, and modulation speed, which are among the most important parameters for modulators. The IL and ER can be obtained from the formulas $IL = 10\log ([P_{out,on} /P_{in} ]_{{\lambda = \lambda_{0} }} )$ and $ER = 10\log ([P_{out,on} /P_{out,off} ]_{{\lambda = \lambda_{0} }} )$, respectively.

To obtain the bandwidth of the 3 dB cross-section, a low-pass filter can be used. The energy consumption per bit can be computed as 1/4 CV^2^. The values of key parameters for the two modulators obtained from the above relations are presented in Table 1.

**Table 1:** The characteristic of two first and second type modulators each channel.

Modulator	Ins. loss (dB)	Ext. ratio (dB)	Modulation voltage (V)	Modulation speed (GHz)	Energy consumption (fJ/bit)	Device footprint (µm^2^)
Type.1	0.027	3.484	0.3	119.89	0.59*	1.892
Type.2	0.035	4.83	2.3	38.67	21.77	0.186

*aJ/bit (a = 10^−18^).

## Conclusion

In this study, we presented a novel approach for implementing a silicon-on-chip integrated 4 × 1 wavelength division multiplexing (WDM) multiplexer. To the best of our knowledge, this was the first time that a multiplexer was based on an array of electro-optical modulators combined with crosstalk cancellation techniques. Each channel of the multiplexer consisted of two electro-optic modulators. The first modulator was responsible for extracting the desired wavelengths used in WDM technology and utilized a 1D-PhCNBC structure. By adjusting the carrier concentration of the Indium Tin Oxide (ITO) material through voltage application and incorporating defects in the mirror sections, we were able to efficiently extract all WDM wavelengths from the input spectrum. The second modulator, based on hybrid plasmonics, functioned as a switch to remove crosstalk. By either allowing the passage of light (on state) or blocking it (off state), it effectively eliminated interference between adjacent channels. To achieve the off state and enter the epsilon-near-zero (ENZ) region, higher voltages were applied to increase absorption in the modulator. The key parameters of both modulators for each channel were summarized in a table in the previous section. Overall, the proposed design offered compactness and high integration capabilities, making it suitable for the development of optical chips used in optical telecommunication systems. By combining the advantages of electro-optical modulators and crosstalk cancellation techniques, we anticipated that our proposed design would contribute to the advancement of WDM multiplexing technology and facilitate the implementation of efficient and compact optical communication systems.

## Data Availability

The data that support the findings of this study are available from the corresponding author upon reasonable request.
